# Potential role of resveratrol and its nano-formulation as anti-cancer agent

**DOI:** 10.37349/etat.2022.00105

**Published:** 2022-10-31

**Authors:** Akshay Kumar, Balak Das Kurmi, Amrinder Singh, Dilpreet Singh

**Affiliations:** 1 Department of Quality Assurance, ISF College of Pharmacy, GT Road, Moga 142001, Punjab, India; 2 Department of Pharmaceutics, ISF College of Pharmacy, GT Road, Moga 142001, Punjab, India; NGO Praeventio, Estonia

**Keywords:** Resveratrol, cancer, solubility, nano-formulations, synergism

## Abstract

The uncontrolled and metastatic nature of cancer makes it worse and more unpredictable. Hence, many therapy and medication are used to control and treat cancer. However, apart from this, many medications cause various side effects. In America, nearly 8% of patients admitted to the hospital are due to side effects. Cancer is more seen in people residing in developed countries related of their lifestyle. There are various phytoconstituents molecules in which resveratrol (RSV) is the best-fitted molecule for cancer due to its significantly less adverse effect on the body. RSV inhibits the initiation and progression of cell proliferation due to the modulation of various pathways like the phosphoinositol 3 kinase (PI3K)/protein kinase B (AKT)/mammalian target of rapamycin (mTOR) pathway. RSV downgraded cell cycle-regulated proteins like cyclin E, cyclin D1, and proliferating cell nuclear antigen (PCNA) and induced the release of cytochrome c from the mitochondria, causing apoptosis or programmed cell death (PCD). A great benefit comes with some challenges, hence, RSV does suffer from poor solubility in water i.e. 0.05 mg/mL. It suffers from poor bioavailability due to being highly metabolized by the liver and intestine. Surprisingly, RSV metabolites also induce the metabolism of RSV. Hence, significantly less amount of RSV presented in the urine in the unchanged form. Due to some challenges like poor bioavailability, less aqueous solubility, and retention time in the body, researchers concluded to make the nanocarriers for better delivery. Adopting the technique of nano-formulations, increased topical penetration by up to 21%, improved nano-encapsulation and consequently improved bioavailability and permeability by many folds. Hence, the present review describes the complete profile of RSV and its nano-formulations for improving anti-cancer activity along with a patent survey.

## Introduction

Cancer is a genetic disease caused by various factors like DNA mutation, including radiation that may be ultraviolet radiation, and some environmental factors, alteration in cell division [1]. Cancer is more predominant in developed countries where their living standards are high, like the United States of America (USA), where approximately 1.9 million patients are diagnosed with cancer, and about 609,360 deaths of cancer in 2022 [2]. The most common cancer is in the lungs, prostate, and colorectal cancer in about 43% of total cancer in males. However, in females, the predominant cancer is breast, lung, and colorectal in about 50% of total cancer in female surveys in 2020, according to the national cancer institute, USA. The mortality due to cancer is higher in males (189.5 per 100,000) than in women (135.7 per 100,000) [3]. Cancer treatment contains one or a combination of methods used to treat or minimize the severity of symptoms like biomarker testing for cancer treatment, chemotherapy, hormone therapy, radiation therapy, immunotherapy, and surgery. Natural drugs benefit from synthetic drugs because they have fewer side effects, especially when cancer is concerned. In America, 8% of patients are admitted to hospitals due to the side effects of synthetic drugs [4].

**Figure 1.**
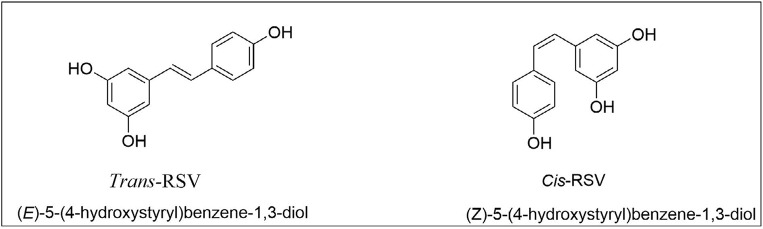
Chemical structure of RSV in the *trans* and *cis* form

Resveratrol (3,5,4’-trihydroxy-*trans*-stilbene, RSV) is obtained from various plant-based sources and also obtained from synthesis and recombinant technology. It was first isolated in 1939 from *Veratrum grandiflorum* O. Loes [5]. RSV was found to manage various disease conditions due to its binding to different enzymes and proteins of various pathways, which regulate the gene expressions or modulate the activity of other processes necessary for its effect. Apart from all its beneficial values for human health, it has some challenges, including extensive bioavailability and metabolism by the enzymes in the liver and intestine and undergoing phase 2 metabolism [6]. RSV has very effective properties to have an anti-cancer agent [7]. It also has other properties, which are discussed in Figure 1. Nanocarriers have advantages when it comes to delivering the drug to the targeted site or, in general, with improved bioavailability and therapeutic efficiency of the drug due to the accumulation of the drug at a particular site [8]. Nanocarriers comprise various nanomaterials that transport chemicals [active pharmaceutical ingredient (API)] to various body parts. Nanocarriers enable fascinating advantages over the conventional delivery system. This system increases *in vivo* efficiency by targeting drug delivery to the specific site, such as the cellular level due to the targeting ligand attached to the surface to nanocarriers, favorable biodistribution, enhanced intracellular penetration, and drug delivery circulates a long time in blood [9]. All these characteristics make the nanocarriers a perfect candidate for chemotherapy. Nanoformulation plays a crucial role. In this case, RSV has very low solubility and bioavailability which the nanoformulation improves to some extent. Hence, the present review gives a brief art of RSV as an anti-cancer agent, its associated formulations for improving therapeutic activity, and a patent survey of new technologies.

## RSV: an overview

RSV is obtained from natural resources of red grapes, blueberries, raspberries, mulberries, cranberries, red wine, nuts, and peanuts [10]. RSV is extracted from wild *Polygonum cuspidatum*’s root [11]. RSV can also synthesize in the laboratory by the Witting reaction and decarbonylative Heck reaction [12]. RSV occurs in two forms, *cis*-RSV and *trans*-RSV (Figure 1), in which *trans*-isoforms are easily converted into *cis*-isoforms (*cis*-RSV) by heat and ultraviolet radiation [13]. Using recombinant technology, RSV is produced by yeast (*Saccharomyces cerevisiae*) with the help of glucose and ethanol, which is used in the cosmetic ingredients and food supplement industry [14]. It is a chemical stilbenoid (polyphenolic, phytoalexin) released by various plants during stress or injury during a pathogen attack (bacteria, fungi) [15]. Moreover, *trans*-RSV effectively managed cognitive disorders like attention deficit hyperactivity disorder (ADHD), Alzheimer’s, and dementia [16].

**Figure 2.**
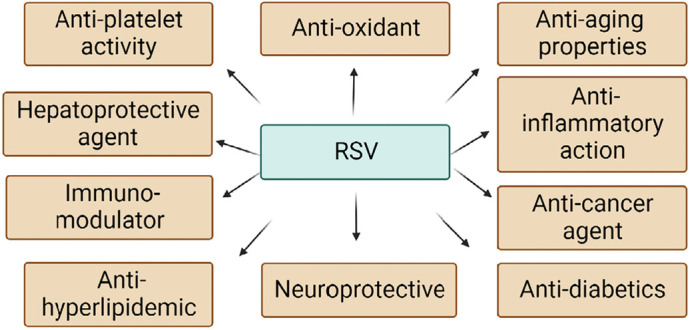
Biological effects of RSV

RSV has the chemical formula: C_14_H_12_O_3_, molecular weight: 228.24, melting point: 254, boiling point: 449.1 ± 14, Log P: 3.10 and solubility in water is 0.05 mg/mL [17] and in polyethylene glycol (PEG) 400 is about 374 mg/mL [18]. RSV consists of phytoestrogen similar to estradiol, a major constituent of estrogen. RSV caused alteration in almost 127 pathways, polyacrylamide gel electrophoresis (PAGE) analysis says. It includes glycolysis, Krab’s cycle [tricarboxylic acid (TCA) cycle], electron transport chain, oxidative phosphorylation, insulin signaling, and sterol biosynthesis. RSV protects and enhances living standards by having anti-cancer, anti-oxidant, anti-inflammatory, and neuroprotective properties [19]. RSV is highly lipophilic. It comes under biopharmaceutical classification system 2 (BCS2) [20]. It can cross the membrane and act on the brain by entering the blood-brain barrier (BBB) [21]. Due to its low pharmacokinetic properties, such as solubility in an aqueous medium (0.05 mg/mL), highly metabolized and excreted by the body, it makes highly inefficient oral doses. These doses form overall have very low bioavailability [22]. Oral absorption of RSV is relatively high, about 75%, but the drug rapidly metabolizes through first-pass metabolism by the liver and intestine [23]. Due to this concern about the pharmacokinetic improvement of doses form, increase in demand. Nanotechnology plays a significant role in delivering formulation to the demands of pharmacokinetic profiles [24]. Many *in vitro* studies and animal studies found that RSV has the potential to exert a favorable effect on clinical studies [25]. RSV targets various enzymes and proteins such as cyclooxygenases (COXs), lipoxygenases, kinases, sirtuins (SIRTs), ribonucleotide reductase, and DNA polymerase to show biological action [26]. The major activities of RSV are given in Figure 2.

### RSV: biopharmaceutical challenges associated with different routes of administration

There are many challenges with RSV delivery using various routes. RSV possesses low solubility in an aqueous medium and almost zero bioavailability profile which is not fit for oral and topical applications alone. That is where novel drug delivery systems play an essential role. Although it is a perfect anti-oxidant, chemoprotective and anti-inflammatory agent [27], problems arise due to insolubility, less skin penetration, poor photostability, and bioavailability.

**Figure 3.**
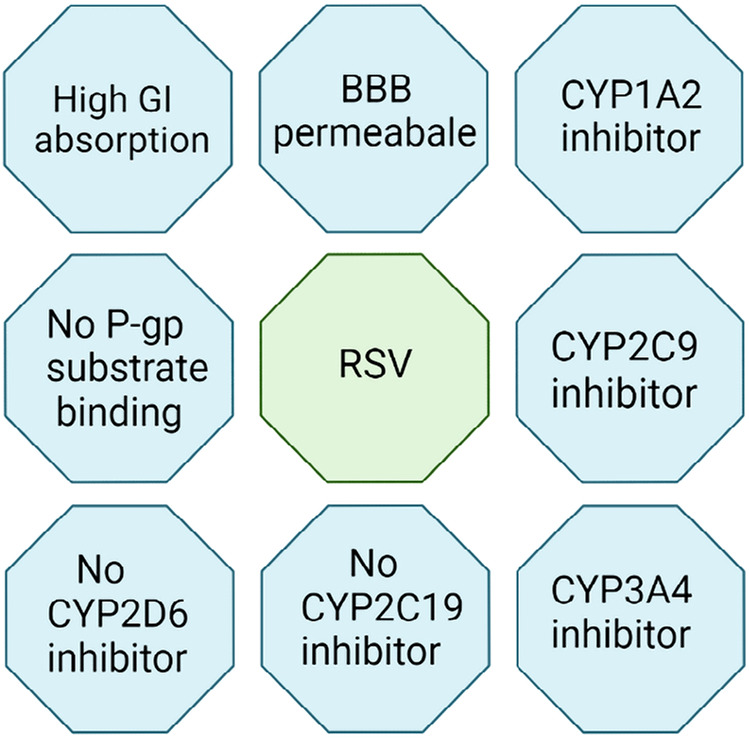
Pharmacokinetic properties of RSV. CYP1A2: cytochrome P450 1A2; P-gp: P-glycoprotein; CYP2C9: cytochrome P450 2C9; CYP2D6: cytochrome P450 2D6; CYP3A4: cytochrome P450 3A4

Oral route—RSV contributes various effects, as discussed above, but comes with challenging problems like low solubility (in water—0.05 mg/mL) and metabolism [17]. RSV quickly absorbs into the portal veins nearly 75% due to its lipophilic nature having Log P/Ko/w is 3.10. On the other hand, it is aggressively metabolized by the liver through phase 2 metabolism and the formation of sulfated, methylated, and glucuronidated complexes. Sulfate complexes with phenolic groups play a rate-limiting step in the bioavailability of RSV. Some of these complexes come back to the gastrointestinal tract (GIT) to induce metabolism [6]. It is fascinating to know that RSV itself increases the metabolism of its own. A significantly less amount of drug passes to the systemic circulation in unchanged form, but most drugs make complexes with albumin and lipoprotein. It also enabled passive diffusion and temperature-mediated diffusion. However, most drugs combined with albumin complexes are responsible for the drug’s transportation and distribution to the various compartment for cellular uptake [28]. The active metabolite of RSV also has the activity of anti-cancer and others (Figure 3). The study indicates that after 25 mg, oral administration of doses shows somewhere 70% absorption and peak plasma level of 491 ± 90 ng/mL, and a half-life of 9.2 ± 0.6 h. Most of the doses are found in the urine, which liquid chromatography-mass spectrometry (LC-MS) detects. RSV is easily uptaken by epithelial cells. Hence the concentration of drugs in these cells is high [29].

Topical route—A marked topical therapy requires excellent apparent permeability across the skin while showing less skin irritation. The topical application of RSV is limited because of its low permeability and poor skin retention [30]. This causes a decrease in the concentration of API required for efficacy, and less amount of drug reaches the target site. With molecular weight > 500 Da and a high partition coefficient, RSV fails to penetrate the stratum corneum. Furthermore, RSV is also associated with mild allergic reactions and erythema, which causes poor patient compliance. Hence, the lacks of effective delivery of RSV and undesirable skin interactions are the main reasons for its poor therapeutic efficacy.

Parenteral route—Transition points, where pharmaceutical errors are more likely to occur, require specific attention for safe medication delivery. Compared to other drug delivery methods, parenteral administration is favored, such as in cases of cardiac arrest and anaphylactic shock [31]. This method of administration has many benefits, including avoiding first-pass metabolism, improved bioavailability, and consistent dose. Parenteral delivery, which is more controlled than oral administration in terms of dose and pace, results in more predictable pharmacodynamic and pharmacokinetic characteristics. As a result of the serious danger that could result from improper usage, several parenteral pharmaceuticals are categorized as high-alert drugs [32]. However, the parenteral administration of RSV causes severe adverse reactions like anaphylactic shock, muscle spasms, mild inflammation, etc. During multiple dosing, steady-state concentrations are unable to maintain leading to repetitive dosing leads to poor patient compliance.

### How RSV beneficial for cancer patients

To show its effect, RSV exerts an effect on the various pathways, receptors, and other modulators. However, the causes of cancer are still unclear, but some hypothesis includes cytoplasmic cell division, abnormal cell, and gene hypothesis. RSV activates the SIRT1 [31], an enzyme located in the cell nucleus responsible for the deacetylation of histone and non-histone proteins, including transcription factors [32]. It is also required to regulate the various pathways that affect circadian rhythms, endothelial function, inflammation, immune function, metabolism, cell survival, and stress resistance. In cancer, SIRT1 regulates abnormal metabolic control, defects in the cell cycle, and inflammation [33]. It also regulates other conditions like cardiovascular diseases, obesity, and neurodegenerative diseases. RSV modulates the nuclear factor-κB (NF-κB) pathway by inhibiting the proteasome in human articular chondrocytes [34], which regulates the protein responsible for apoptosis and cell cycle progression. NF-κB regulates the antiapoptotic gene [35]. The active form of NF-κB results in the expression of a gene responsible for cell proliferating and protecting cells from apoptosis [programmed cell death (PCD)]. Whereas defects in the NF-κB result in increased apoptosis. NF-κB alters the activity of the caspase family enzyme, which is responsible for most apoptotic processes. RSV inhibits interleukin 1 (IL-1)-induced apoptosis simulation of caspase-3 and poly(ADP-ribose) polymerase (PARP) cleavage in human articular chondrocytes [36]. RSV suppressed the NF-κB regulated gene products such as matrix metalloproteinase-2 (MMP-2), MMP-3, MMP-9, vascular endothelial growth factor (VEGF), COX-2 and also inhibited apoptosis [B-cell lymphoma-extra large (Bcl-xL), B cell lymphoma protein-2 (Bcl-2), tumor necrosis factor-α (TNF-α) factor 1] [37].

Suppression of MMP-2 lead to the inactivation of stress-activated protein kinase/c-Jun N-terminal kinase (SAPK/JNK) and p38 mitogen-activated protein kinases (MAPK) signaling pathway, which causes inhibition of A549 cell invasion beneficial for epithelial carcinoma. RSV inhibits the COX enzyme, which stops prostaglandin formation from arachidonic acid. Prostaglandin is responsible for synthesizing an inflammatory compound that promotes tumor cell proliferation or tumorigenesis. RSV inhibits the insulin-like growth factor-1 receptor (IGF-1R)/Akt/Wingless and int-1 (Wnt) signaling pathways that activate tumor protein (p53), leads to the suppression of colon cancer cell proliferation and induces apoptosis [38]. PI3K/Akt/mTOR pathway regulates various cellular activities such as cell proliferation and differentiation, cellular growth, survival, and mobility [39]. Components of these pathways show several abnormalities during various tumor growth, making it an exciting target for anti-cancer therapy. RSV inhibits PI3K/Akt/mTOR pathways, which are combined with other therapy [40]. RSV also downgraded cell cycle-regulated proteins such as retinoblastoma (Rb), cyclin-dependent kinase 2 (CDK2), CDK4, cyclin E, cyclin D1, and proliferating cell nuclear antigen (PCNA) [41], which inhibits the Akt pathways, causing the death of bladder cancer cell, liver cancer cell, and rats aortic vascular smooth muscle cell. RSV induces cell death, inhibits cell proliferation, and affects cell progression in the ovarian cancer cell line (A2780 cell) [42]. RSV induces the release of cytochrome c from the mitochondria to cytosol, which bind to apoptotic protease activating factor 1 (Apaf-1) to cause caspase activation and cause apoptosis or PCD. RSV also induces auto-phagocytosis, and the Bcl-2 and Bcl-xL do not inhibit the action of RSV in ovarian cancer cell A2780 [43].

### Nano-carrier: a critical appraisal

A nanomaterial employed as a transport component for another chemical, such as a drug, is called a nanocarrier [44]. Micelles, polymers, carbon-based materials, liposomes, and other substances are frequently utilized as nanocarriers. Nanocarriers’ distinctive properties suggest a potential utility in chemotherapy, and they are now being investigated for their use in medication delivery [45]. Since micro-capillaries have a diameter of 200 nm and a diameter range of 1–1,000 nm for nanocarriers [46], nanomedicine frequently refers to objects with a diameter of less than 200 nm. Nanocarriers can deliver medications to parts of the body that would not typically be accessible due to their small size [47]. Because nanocarriers are so tiny, it is frequently challenging to administer substantial pharmacological doses using them. The low drug loading and drug encapsulation that frequently results from the emulsion procedures used to create nanocarriers presents a challenge for therapeutic application. Polymer conjugates, polymeric nanoparticles, lipid-based carriers, dendrimers, carbon nanotubes, and gold nanoparticles are a few of the newly identified nanocarriers [48]. Liposomes and micelles are two types of lipid-based carriers. Gold nanoshells and nanocages are two types of gold nanoparticles. Hydrophobic and hydrophilic medications can be distributed throughout the body thanks to the employment of several types of nanomaterial in nanocarriers. Since the human body is mainly made up of water, one important therapeutic advantage of nanocarriers is their capacity to transport hydrophobic medications to people successfully. Depending on the orientation of the phospholipid molecules, micelles can contain either hydrophilic or hydrophobic medicines [49]. Some nanocarriers have nanotube arrays that enable them to hold medications that are both hydrophilic and hydrophobic. Unwanted toxicity from the sort of nanomaterial being employed is one potential issue with nanocarriers. If inorganic nanomaterial builds up in specific cell organelles, it can also be hazardous to humans. To create safer, more efficient nanocarriers, new research is being done. Since protein-based nanocarriers are found in nature and often exhibit lower cytotoxicity than synthetic compounds, they hold promise for use as therapeutic agents [50, 51].

Because they can deliver pharmaceuticals to site-specific targets, nanocarriers are helpful in the drug delivery process because they allow drugs to be administered in some organs or cells but not in others [52]. Site-specificity is a significant therapeutic advantage since it stops medications from being administered to the incorrect locations. Nanocarriers have promise for application in chemotherapy because they can lessen the chemotherapy’s harmful, widespread toxicity on the body’s healthy, rapidly dividing cells. Chemotherapy medications must be given to the tumor without spilling over into healthy tissue because they can be highly damaging to human cells.

### RSV nano-formulations

#### Solid lipid nanoparticles

Solid lipid matrices with surfactants display the best *in vivo* tolerability, good physico-chemical stability, and prolonged drug release. Solid lipid particles are made up of particles in the micrometer size range. RSV-loaded solid lipid nanoparticles (SLNs) show a better ability to suppress or inhibit the growth of human breast cancer cells (MDA-MB-231 cells) in comparison with free RSV [53]. Compared to liposomes, they have been described as lipidic drug carrier systems for topical applications that can replace polymers and enable large-scale manufacture at a relatively lower cost [54]. Solid lipid microparticles (SLMs) for RSV have the potential for oral delivery to improve their solubility and bioavailability, although having received less research attention than SLNs for skin applications. Several techniques, including solvent evaporation, melt dispersion, hot and cold homogenization, spray drying, and spray congealing, can be used to create SLMs [55]. D-*α*-tocopheryl polyethylene glycol 1000 succinate (TPGS) coated SLNs formulation of RSV given intravenously showed 9.37 folds higher plasma half-life and improved circulation time, better passive brain delivery of drug in Charles Foster rats [56].

#### Microemulsions

Because of their cutaneous tolerance and balanced hydrophilic-lipophilic character, microemulsions (MEs) are clear, colloidal, isotropic, and thermodynamically stable liquid dispersions of oil and water [57]. A multiphase system made up of water, oil, a surfactant, and a cosurfactant like alcohol that primarily serves as a co-solvent and demonstrates transparency by supplying globule size below 140 nm was the first to be referred to as a ME by Schulman in 1959 [58, 59]. A study shows that MEs gel-loaded RSV for sustained release in *in vitro* and *ex vivo* show 71.11 ± 0.47 and 68.15 ± 0.12 respectively in a 24 h period, which are tested for B16F10 melanoma cell lines [60]. MEs provide various benefits for topical distribution, including the capacity to dissolve lipophilic medicines effectively, improved skin permeability, and a longer release of both lipophilic and hydrophilic medications [57]. According to reports, oleic acid (OA) MEs exhibit a greater capacity for solubilizing RSV and a greater concentration of drug retention in the skin [61]. MEs have a high capacity for drug loading, and because they have a high capacity for solubilizing drugs [62], they can get through the stratum corneum barrier and partition the medication into the skin [63, 64]. A study indicated that MEs protect the RSV from ultraviolet B (UVB) radiation for up to 1 h and delay photodegradation which further helps in uptakes of RSV in the skin [65].

#### Vesicular drug delivery systems

Vesicular drug delivery systems consist of one or more concentric bilayers and are highly organized assemblies [66–68]. They are created when amphiphilic building components self-assemble in the presence of water. These systems can localize the medication to the site of action, reducing the concentration of the drug at other places in the body, and making them effective for targeted drug delivery [69]. When compared to other non-vesiculized dose forms, this one help to achieve improved skin permeability and retention, as some of the popularly studied. Liposomes, transferosomes, ethosomes, and niosomes are examples of vesicular systems for topical delivery [70, 71].

#### Liposomes

An aqueous core is encircled by a hydrophobic lipid bilayer membrane that contains phospholipids and cholesterol in liposomes [72], which are biocompatible and biodegradable vesicles. They allow for better active ingredient absorption through the skin. Although they are simple to prepare, they are prone to structural failure and oxidation. The design, content, size, and drug-release properties of liposomes are flexible [73, 74]. According to published clinical research, liposomal anticancer medication has less toxicity and better tolerance [75]. For *in vitro* skin permeation and *in vivo* antineoplastic effect [76], for example, in combination with RSV and 5-fluorouracil (5-FU) in a liposome shows better skin cancer activity as compared to a single drug [77]. A study conducted by researchers concluded that RSV-loaded liposomes show stable, good loading efficiency (70–75%), and prolonged release *in vitro* which improved the activity of anti-proliferation and apoptosis in U-87 MG cell line and xenograft-bearing mice [78]. Another study shows that 4-Carboxybutyl triphenylphosphonium bromide (TPP)-based liposomes showed better mitochondrial targeting and enhance the activity of RSV in B16F10 cell lines [79].

#### Transferosomes

Transferosomes are extremely flexible, self-assembled, and ultradeformable vesicles with an aqueous core and a complex lipid bilayer on each side [80]. These vesicles are self-regulating and self-optimizing because of their structure and composition. Transferosomes can spontaneously penetrate the stratum corneum (SC) because they can efficiently cross a variety of transport barriers [81]. They have better efficacy in sustained release applications for topical medication administration and are more elastic than liposomes [82, 83]. A study showed that transferosomes encapsulated RSV has more penetration power (27.5%) in comparison with free RSV [84]. Particle size and shape, zeta potential, viscosity, entrapment effectiveness, deformability, *in vitro* drug release, kinetics, and drug retention were characterization characteristics. The transferosome was created using the solvent evaporation method, whereas the reverse-phase evaporation method was employed to create the liposomes and niosomes.

#### Niosomes

Niosomes are composed of nonionic surfactant vesicles and are similar to liposomes in structure. These formulations are becoming more and more significant for cutaneous drug administration because they have traits such as improved drug penetration, prolonged drug release, increased drug stability, and the capacity to transport both hydrophilic and lipophilic drugs [85]. Pando et al. [86] formulated a niosomes taking surfactant G64, OA, and linoleic acid (LA) as a penetration enhancer and loaded with RSV which was then evaluated. Results are very interesting, the niosomes prepared by ethanol injection modified method (EIM) show a 21% increase in penetration enhancement, *ex vivo* experiment held in Franz diffusion cells collected from newborn pig skin [86]. Different nano-formulations loaded with RSV improving therapeutic activity are listed in Table 1.

**Table 1.** A 10-year literature review of different nano-formulations of RSV

**Route of administration**	**Drug (API)**	**Excipient**	**Formulation**	**Observations**	**Reference**
Parenteral delivery (intravenous)	*trans*-RSV	TPGS	SLN	Showed 9.37 folds higher plasma half-life and improved circulation time, better passive brain delivery of drug in Charles Foster rats.	[56]
Oral delivery	RSV	Magnesium dihydroxide	Solid dispersion	Improved solubility and bioavailability (3.3-fold) as compared to RSV alone.	[87]
		A mixed lipid phase (castor oil/Capmul MCM 1:1) and a mixed surfactant phase (Kolliphor EL/Kolliphor RH 40 1:1)	Self-micro emulsifying drug delivery system (SMEDDS)	Formulation improved the bioavailability and reduce toxicity which is further used in the supplement as well as the pharmaceutical industry.	[88]
		poly (DL-lactide-co-glycolide) (PLGA)	Systematically optimized nanoparticles	Ka and AUC are 7.17 and 10.6 folds respectively while bioavailability showed 2.78 folds.	[89]
		Phospholipids, Sylysia 350, TPGS	Solidified phospholipid-TPGS	Increased bioavailability by dissolution rate and absorption.	[90]
		Zein	Nano capsulation	Improved bioavailability and permeability of 1.15 folds.	[91]
		Di stearoyl phosphatidyl choline, sodium taurocholate, cholesterol	Proliposomal formulation	AUC and C_max_ increased by 2 folds.	[92]
Nasal delivery	RSV	8.75% w/v chitosan solution, SLMs, chitosan	LMs uncoated or coated with chitosan	Improve C_max_ (60 min) about 9.7 ± 1.9 μg/mL. Effective targeting of brain drug delivery by nasal route.	[93]
		Transferosomes	Intranasal transferosome mucoadhesive gel	Improved C_max_ and AUC by 2.15 and 22.5 folds respectively as compared to RSV suspension alone.	[94]
Topical delivery	RSV	Gelot 64 (surfactant), OAs and LAs	Niosomes	Topical penetration increased up to 21%.	[86]
		Dendrimer	Dendrimer nanotechnology	78.06% of the formulation showed intradermal permeation and only 37.33% RSV alone.	[13]
		Eugenol	Nano emulsion	The highest RSV penetration into the stratum corneum is about 9.55 (ratio compared to saturated RSV).	[95]
		Limonene	Nano emulsion	The highest permeation of drug to the skin is about 12.61 (ratio compared to saturated RSV).	[95]

AUC: area under the curve; C_max_: peak plasma concentration; LMs: lipid microparticles

### Patent survey of RSV nanoformulation

However, many patents have been filled in the last two decades, which shows a significant interest in this area. Many challenges have been addressed, and various methods have continuously improved product quality by innovation in formulating RSV. Some of them are nanoformulation which improves severe problem that occurs during the drug delivery of RSV. A list of such patented formulations and methods is given in Table 2.

**Table 2.** Patent survey of different nano-formulations of RSV

**Patent number**	**Inventor name**	**Invention**	**Details**	**Reference**
WO-2012017451-A1	Pratibha Omray, Vinay Kumar Tripathi	A bio-stabilized resveratrol formulation	A herbal medicament contains 50–80% RSV and other ingredients which helps to prevent and treat cancer, Alzheimer’s disease, obesity, and others.	[96]
US20110281957A1	Eric Kuhrts	Enhanced bioactive formulations of resveratrol	Enhanced solubility of RSV which is used for the treatment of disease states like cancer.	[97]
ES2798403T3	Dariush Behnam, Marshall A Hayward	Resveratrol solubilization product for pharmaceutical purposes	RSV solubilization product is used for pharmaceutical purposes and helps increase plasma level for therapeutics effects and also reduce GIT side effects as compared with natural RSV.	[98]
WO2001030336A2	John M. Pezzuto, Richard C. Moon, Mei-Shiang Jang, Aomar Ouali, Shengzhao Lin, Karla Slowing Barillas	Pharmaceutical formulations comprising resveratrol and use thereof	Designed the formulation for topical delivery which are helpful in preventing and treating the skin disorder caused by inflammation, sunburn, and aging.	[99]
WO2010059628A1	Arthur S. Polans, Lalita Subramanian, Ronak Vakil, Glen S. Kwon	Water-soluble formulations of resveratrol and uses thereof	Formulation of RSV with solubilizer poloxamer 334 and the solution can be used for the medicament of cancer.	[100]
CN104688715B	Wang Zhicheng, Zhu Kexin, Wang Bing, Cai Feng, Ren Jianlin, Zhang Tong, Zhang Qi, Zong Shiyu	A kind of resveratrol solid lipid nano granule and preparation method thereof	This invention helps to reduce particle diameter, drug loading and bioavailability is high and has fast absorption, and is convenient to take.	[101]
CN105126116B	Xiao Chunsheng, Chen Xin, Ding Jianxun, Zhuang Xiuli, Chen Xuesi	A kind of resveratrol nano particle and preparation method thereof	The formation of nanoparticle cross-linked structure with RSV increased water solubility and improved stability.	[102]
CN101214225A	Ouyang Wuqing, Yang Baoping	Resveratrol nano emulsion anti-cancer medicine	RSV nano emulsion increase half-life and stability and also prevent RSV to get oxygenated.	[103]

### Future challenges

There is a number of RSV studies that are listed on ClinicalTrials.gov, the database of publicly and privately financed human clinical research. Numerous of these trials have been conducted to assess RSV’s pharmacokinetics, bioavailability, safety, and tolerability. Only a few of the studies mentioned are concerned with determining whether RSV is effective in treating particular malignancies [104]. *Trans*-RSV, *Polygonum cuspidatum* (Japanese knotweed) extract, SRT501 (micronized RSV), RSV-rich seedless red grapes/grape juices (muscadine grapes), and micro-encapsulated RSV are some of the types of RSV used in these studies. Numerous cancer types, including multiple myeloma, breast cancer, follicular lymphoma, and neuroendo-crine tumors are the subject of these trials, although the majority of them examine how RSV affects the growth of colon malignancies. In reality, RSV has shown to be slightly effective in colon cancer clinical trials, which may be related to RSV’s potential direct contact and extended exposure to colonic tissues [105]. The gut epithelium also appears to be well-suited for absorbing nutrients and active chemicals from food and food components [106].

According to ClinicalTrials.gov, it is exciting to note that while planning the new clinical trials, researchers used what they had learned from earlier trials, which should provide improved outcomes. Two of the four ongoing trials are aimed at gastrointestinal malignancies. The goal of one of these trials (NCT00433576) is to determine the ideal RSV dosage that will produce bioactive levels in the colon mucosa. This is significant because an ideal dose has not yet been identified despite extensive RSV studies on colon cancer [106]. Additionally, the researchers will determine whether there are correlated levels of COX-2 and pyrimido[1,2-α]purin-10(3H)-one (M1G) adduct in cancer tissues to examine the mechanistic consequences of RSV treatment of colorectal adenocarcinoma. Given that both of these molecules have been proven to be regulated in colon cancer, this might offer some relevant information. The goal of the other ongoing colon cancer experiment (NCT00578396), which uses seedless red grapes, is to ascertain the highest dietary RSV levels possible. This may be crucial for colon cancer prevention because it would be simple to develop new dietary guidelines for the general public. These trials ought to yield some information that will be beneficial in more in-depth human research that aims to make RSV available in clinics for the treatment of diseases. Diets high in RSV may also be encouraged for improved health and illness prevention [107, 108].

## Conclusions

The vast range of applicability of RSV makes it a center of attraction for the investigation and search for the novelty to improve pharmacokinetics such as (solubility and bioavailability) and toxicological effects on humans. Several nanoformulation such as niosomes, liposomes, MEs, SLNs, nanocapsules, and other formulations enhanced the therapeutic efficacy and solubility of RSV, also improving the bioavailability of oral and increasing retention and penetration of topical formulation many folds, which are discussed in Table 1. Although RSV has many beneficial effects on the body most highlighted are anti-oxidant and anti-cancer activity. It regulates various pathway inhibition (PI3K/Akt/mTOR, IGF-1R/Akt/Wnt, IL-1 induced apoptosis simulation), modulation (NF-κB), and activation (SIRT1) and induction (release of cytochrome c, auto phagocytosis) to exert its effect. Hence, some patent formulations could be a sign of interest. Nevertheless, apart from anti-cancer activity, its applicability is not limited due to its uses in nutraceuticals, herbal and pharmaceutical industries.

## Abbreviations

AKT: protein kinase B
API: active pharmaceutical ingredient
COXs: cyclooxygenases
MEs: microemulsions
MMP-2: matrix metalloproteinase-2
mTOR: mammalian target of rapamycin
NF-κB: nuclear factor-κB
OA: oleic acid
PI3K: phosphoinositol 3 kinase
RSV: resveratrol
SIRTs: sirtuins
SLMs: solid lipid microparticles
SLNs: solid lipid nanoparticles
TPGS: D-*α*-tocopheryl polyethylene glycol 1000 succinate

## Declarations

### Copyright

© The Author(s) 2022.
